# Sterilisation of Surgical Instruments by Formaldehyde

**Published:** 1898-04-30

**Authors:** 


### STERILISATION OF SURGICAL INSTRUMENTS BY FORMALDEHYDE.

In the *Johns Hopkins Hospital Bulletin* a supplementary report appears, by Dr. Reik, of an investigation in regard to the sterilisation of surgical instruments by formaldehyde. The instruments are arranged on wire trays in a small cupboard, in which there is also enclosed a Schering lamp like the Alformant Disinfecting Lamp, by which formaldehyde is evolved from pastiles. The conclusion is arrived at that by this method we have a rapid, cheap, easy and sure method of sterilising instruments without in any way injuring them. So far as we can see the ordinary method of sterilising by boiling in soda solution answers every purpose. Still, there is no doubt that it is a great nuisance having to boil up and afterwards dry all sorts of instruments which may not be required in the operation at all; and if formaldehyde sterilisation will serve the same purpose, leaving the instruments fit to put away without further manipulation, it will be a great convenience. We are not, however, quite convinced. Formaldehyde vapour may be very well so far as regards the surfaces to which it gets access; but in various intricate surgical instruments there are corners and queer places in which surgical dirt may dry up and never be discovered, either by the hand of theatre sister, or by the vapour of the steriliser. All these matters are, however, rendered innocuous by boiling. It is troublesome, no doubt, but we are well repaid by the certainty of its action.

